# Effectiveness and safety of aromatherapy massage for knee osteoarthritis: A protocol for systematic review and meta-analysis

**DOI:** 10.1097/MD.0000000000029039

**Published:** 2022-03-18

**Authors:** Tingting Pang, Chang Liu, Jiahui Li, Junjie Yao, Zhongxu Li, Siyuan Lei, Jiangchun Zhang, Xuefei Li, Li Dong, Yufeng Wang

**Affiliations:** ^a^ *Department of Acupuncture and Tuina, Changchun University of Chinese Medicine, Changchun, China,* ^b^ *Department of Tuina, the Affiliated Hospital to Changchun University of Chinese Medicine, China,* ^c^ *Department of Chinese Medicine and Orthopedics, Zhejiang University of Traditional Chinese Medicine, China,* ^d^ *Department of Rehabilitation Medicine, Changchun University of Chinese Medicine, Changchun, China.*

**Keywords:** aromatherapy massage, knee osteoarthritis, meta-analysis, systematic review

## Abstract

**Background:**

The purpose of this study was to evaluate the effectiveness and safety of aromatherapy massage in the treatment of knee osteoarthritis.

**Methods:**

To collect relevant literature, we will research following databases: PubMed, Web of Science, Scopus, Cochrane Library, Embase, China National Knowledge Infrastructure, China Science and Technology Journal Database and Wanfang Data the time is from inception to December 30, 2021, and the language is limited to Chinese and English. In addition, we will retrieve other literature resources, including the Chinese Clinical Trial Register, conference papers. Two reviewers will independently complete the literature screen and data extraction, and quality assessment of the included studies will be independently completed by 2 other researchers. The primary outcomes included the Western Ontario and McMaster Universities Osteoarthritis Index scale, the visual analog scale, symptom score, Lysholm knee scoring scale, adverse events, and adverse reactions as secondary outcomes would be assessed. RevMan V.5.4.1 software will be used for meta-analysis, and the Grading of Recommendations Assessment, Development and Evaluation (GRADE) will be used to assess the quality of evidence.

**Results:**

This systematic review will be showed a high-quality synthesis to evaluate the efficacy and safety of aromatherapy massage in the treatment of knee osteoarthritis, providing reference for the safe and effective treatment of knee osteoarthritis.

**Conclusion:**

This study provides evidence of whether aromatherapy massage is effective.

Systematic review registration: INPLASY202210010

URL: https://inplasy.com/inplasy-2022-1-0010/

## 1. Introduction

Knee osteoarthritis (KOA) is a chronic degenerative disease that severely affects the quality of life of middle-aged and elderly people. Typical symptoms of KOA include knee pain and tenderness, swelling, stiffness, bone friction sounds (sensation), limited joint movement, and in severe cases, internal or external knee deformity.^[1]^ Knee pain when walking up and down stairs is the first symptom in many patients with early KOA, and it may be an early sign of KOA.^[2]^ Some studies have shown that KOA has a risk of causing lower limb disability of at least 40% in the elderly,^[3]^ and is considered one of the top ten causes of disability.^[2]^ With the increasing aging of the global population, KOA has become a serious public health problem that needs to be addressed in society today.^[4]^ Currently, there is no effective radical treatment for KOA in clinical practice, and the main goal is to relieve joint pain and improve functional joint symptoms.^[5]^ For patients with KOA, they need a safe, reliable and effective treatment to alleviate their typical symptoms, restore the physiological function of the knee joint, and thus improve their quality of life.

Aromatherapy massage is one of the widely popular complementary and alternative medical interventions, that is, the use of various techniques on the human body surface and meridians or acupuncture points, using aromatic substances as the medium for transdermal delivery of massage therapy, in order to achieve the purpose of strengthening the body and treating diseases.^[6]^ Some studies have shown that aromatherapy massage can reduce pain, fatigue, anxiety, and sleep disorders.^[7-9]^ And now clinical studies have proved that aromatherapy massage can reduce pain, improve functional status and enhance the quality of life for KOA patients.^[10]^ After our preliminary study, we found that there is no systematic review on whether aromatherapy massage is safe and effective for patients diagnosed with KOA. Therefore, we used this protocol to comprehensively evaluate the efficacy of aromatherapy massage on KOA.

## 2. Methods and analysis

The study was conducted following the guidelines of the Preferred Reporting Items for Systematic Review and Metaanalysis Protocol (PRISMA-P).^[11]^ This study protocols have been funded through a protocol registry. This protocol of the systematic review has been registered on the INPLASY website. Registration: INPLASY202210010.

### 2.1. Inclusion criteria

#### 2.1.1. Types of participants.

All patients should be diagnosed with knee osteoarthritis, and should be older than 18 years of age. However, their race, gender, and education status are not limited. Patients with acute knee trauma, local skin ulcers, and patients with serious primary diseases such as infectious diseases, cardiovascular diseases, liver and kidney function diseases, tumors, immune system, and hematopoietic system diseases should be excluded.

#### 2.1.2. Types of interventions.

The interventions in the experimental group included only aromatherapy massage. It mainly included different single oil massage and compound oil massage, etc. There was no restriction on the method, time, and frequency of massage in the experimental group. Controlled interventions included control groups with no treatment, sham/placebo groups, or other conventional treatments.

#### 2.1.3. Types of studies.

1. Inclusion: We will include only randomized controlled clinical trials of aromatherapy massage for knee osteoarthritis.
2. Exclusion: We will exclude any other literature including nonrandomized clinical controlled trials, retrospective research literature, conference abstracts, case reports, repeated published literature, and literature of information without data.

#### 2.1.4. Types of outcomes

##### 2.1.4.1. Main outcomes.

The primary outcome was scales of The Western Ontario and McMaster Universities Osteoarthritis Index scale.

##### 2.1.4.2. Secondary outcomes.

Secondary endpoints included visual analog scale, symptom score, Lysholm knee scoring scale, and adverse events.

### 2.2. Data sources and search methods

#### 2.2.1. Electronic searches.

We will collect relevant articles by searching the following databases: PubMed, Web of Science, Scopus, Cochrane Library, Embase, China National Knowledge Infrastructure, China Science and Technology Journal Database, and Wanfang Data. All databases will be searched from inception to December 30, 2021, by the following words: Aromatherapy massage, Knee Osteoarthritis, osteoarthritis, degenerative joint disease, degenerative arthritis, Femorotibia, Randomized controlled trial, Controlled clinical trial, RCT, etc. The research strategy for PubMed is shown in Table 1.

**Table 1:** Search strategy used in PubMed.

**No**	**Search items**
#1	osteoarthritis (All Fields)
#2	osteoarthr* (All Fields)
#3	(oa) OR (OA) (All Fields)
#4	degenerative joint disease (All Fields)
#5	degenerative arthritis (All Fields)
#6	#1 OR #2-#5
#7	knee (All Fields)
#8	knee* (All Fields)
#9	Femorotibia (All Fields)
#10	#7 OR #8 OR #9
#11	#6 AND #10
#12	Aromatherapy (All Fields)
#13	Aroma* (All Fields)
#14	Therapy, Aroma (All Fields)
#15	#12 OR #13 OR #14
#16	Massage (All Fields)
#17	Tuina (All Fields)
#18	#16 OR #17
#19	#15 AND #18
#20	Randomized controlled trial (All Fields)
#21	Controlled clinical trial (All Fields)
#22	Randomized (All Fields)
#23	Randomly (All Fields)
#24	#19 OR #20-#22
#25	#11 AND #19AND #24

#### 2.2.2. Searching for other resources.

We will search the reference list of the included studies and existing systematic reviews related to our topic. We will also search for other literature resources, including the Chinese Clinical Trial Register, conference papers, and other related gay literature to make our search as complete as possible.

### 2.3. Data collection and export

Two researchers independently screened the literature according to the eligibility criteria. First, they eliminated duplicate articles using EndNote V.× 9.0, and excluded articles that did not meet the inclusion criteria by reading the title and subject. Second, they will perform a screen again of the remaining articles by reading the full text according to the inclusion and exclusion criteria and determine whether it is available for the systematic review. We will also record the excluded papers and explain the reasons for this; the specific screening process is shown in Figure 1. If there is disagreement during, the third researcher will be invited to make a decision.

**Figure:**
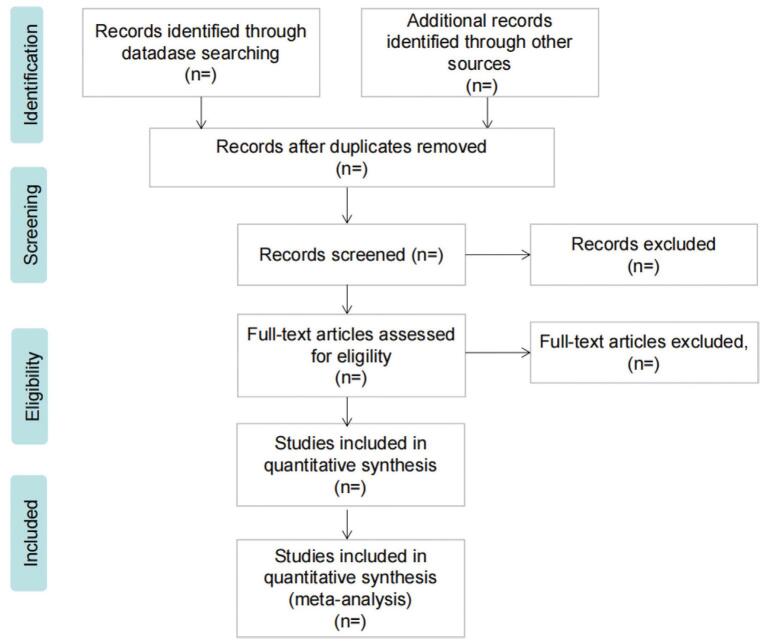
**Figure 1.** Flow diagram of study selection process.

### 2.4. Data extraction and analysis

Data extraction will be performed by two reviewers independently, and the results will be cross-matched. When the differences and opinions are inconsistent, they should be settled through discussion. If the differences encountered cannot be resolved through discussion, a third researcher will be invited to resolve them. We will make an Excel to extract data which includes the first author, country, year of publication, patient characteristics, number of participants, interventions, outcomes, results, main conclusions, conflicts of interest, ethical approval, and other information. If necessary, we will contact the corresponding author by e-mail to obtain more accurate data.

### 2.5. Assessment of risk of bias in the included studies

Two researchers will independently evaluate the bias risk, including studies using the assessment tool of risk bias in the Cochrane Handbook V.5.1.0. The contents included random sequence generation, allocation sequence concealment, blinding of participants and personnel, outcome assessors, incomplete outcome data, selective outcome reporting, and other sources of bias 7 entries. According to the specific criteria of the evaluation manual, the researchers identified the included studies as low risk bias, high risk bias or unclear risk of bias. In the process, if there is disagreement, a third reviewer will be invited to make a decision.

### 2.6. Assessment of heterogeneity

The heterogeneity test will be carried out among all studies included using the *I*^2^ statistic. When *I*^2^ was < 50%, there was no significant heterogeneity. Otherwise, if the result of *I*^2^ is more than 50%, we believe that there is obvious heterogeneity, and subgroup analysis and sensitivity analysis will be conducted to investigate the sources of heterogeneity.

### 2.7. Assessment of reporting biases

We will analyze the quality of publication bias using Rev Man 5.4.1 software in inverted funnel plots and performing Egger test when there were > 10 trials included in the meta-analysis.

### 2.8. Data synthesis

The meta-analysis of data from included outcomes will be performed using the Rev Man V.5.4.1, and we will choose a randomized or fixed effect model for data statistics according to the results of the heterogeneity test. The enumeration data were expressed as relative risk, and the weight mean difference was used as the measurement data; each effect amount was expressed in 95% confidence interval. The specific methods were as follows: If the heterogeneity was low (*I*^2^ < 50%, the fixed-effects model was used for data synthesis. If there is high heterogeneity (*I*^2^ > 50%), the random-effects model will be used for data synthesis after excluding possible heterogeneity sources. The investigation methods included subgroup and sensitivity analyses. If data cannot be synthesized, we provide a descriptive analysis to solve this problem.

### 2.9. Subgroup analysis

If there was high heterogeneity (*I*^2^ > 50%) among the included studies, we conducted a subgroup analysis to analyze the sources of heterogeneity according to the following factors: age, sex, race, courses, sample sizes, different methods of aromatherapy massage, and other possible factors affecting the results.

### 2.10. Sensitivity analysis

To test the stability and reliability of the results of this study, we conducted a sensitivity analysis according to the following points: method quality, sample size, and missing data. After that, we will perform a data analysis again and compare the results. If there was no directional change after the sensitivity analysis, the results were stable.

### 2.11. Grading the quality of evidence

We will use the Grading of Recommendations Assessment, Development, and Evaluation to access confidence in cumulative evidence.^[12]^ The risk of publication, heterogeneity, indirectness, imprecision, and publication bias were assessed, and the results were divided into 4 levels: high, moderate, low, and very low.

### 2.12. Ethics and dissemination

Ethical approval will not be required, as no primary information of individual patients was collected. We will publish this article in a peer-reviewed journal.

## 3. Discussion

Osteoarthritis of the knee is one of the leading causes of disability worldwide and is a serious threat to the physical and mental health of the population.^[13]^ Currently, treatment methods mostly use oral non-steroidal anti-inflammatory drugs as well as motor-behavioral interventions, while some choose physical therapy interventions and, in severe cases, surgery. However, KOA is often lingering and recurrent, and there is no complete cure.^[1]^ Although modern medical treatments are common in the clinical treatment of KOA, there are corresponding complications and risks. Long-term application of non-steroidal anti-inflammatory drugs, intra-articular injections, and chondrotrophic drugs can cause drug resistance, drug dependence, various toxic side effects, and even aggravate the damage to joint cartilage.^[14]^ Surgical treatment is mostly used in the later stages of the disease and has certain treatment limitations, including high cost, high surgical risk, postoperative complications, and uncertain efficacy.^[15]^ Previous studies have confirmed that aromatherapy is an effective treatment method. It helps to improve the pain, stiffness, and functional status of KOA patients.^[7-9]^ However, the evidence for aromatherapy massage for KOA lacks a comprehensive and systematic assessment. The evidence lacks a comprehensive and systematic assessment. Therefore, we hope that this study may provide valuable information for clinical purposes. However, the study may have some potential limitations. First, there may be a large variability due to the different methods of aromatherapy massage. Second, the quality of the study may be compromised as we included only clinical trials published in Chinese or English.

## Author contributions

**Data curation:** Siyuan Lei, Xuefei Li.

**Formal analysis:** Jiangchun Zhang.

**Funding acquisition:** Yufeng Wang.

**Investigation:** Chang Liu, Zhongxu Li.

**Methodology:** Jiahui Li, Junjie Yao.

**Validation:** Tingting Pang, Yufeng Wang.

**Writing** - **original draft:** Tingting Pang.

**Writing** - **review & editing:** Li Dong, Yufeng Wang.
